# Removal of Tannic Acid Stabilizes CuO Nanoparticles from Aqueous Media by PAFC: Effect of Process Conditions and Water Chemistry

**DOI:** 10.3390/molecules26185615

**Published:** 2021-09-16

**Authors:** Rizwan Khan, Muhammad Ali Inam, Kang Hoon Lee

**Affiliations:** 1 Department of Chemical Engineering, Quaid-e-Awam University of Engineering, Science and Technology (QUEST), Nawabshah 67480, Pakistan; rizwansoomro@quest.edu.pk; 2 Institute of Environmental Sciences and Engineering (IESE), School of Civil and Environmental Engineering (SCEE), National University of Sciences and Technology (NUST) H-12 Campus, Islamabad 44000, Pakistan; ainam@iese.nust.edu.pk; 3 Department of Civil and Environmental Engineering, Hanyang University, 222 Seongdong-gu, Seoul 04763, Korea

**Keywords:** organic ligands, tannic acid, coagulation, sedimentation, nanoparticles, polyaluminum ferric chloride

## Abstract

The increased utilization of CuO nanoparticles (CuO NPs) in various fields has raised concerns about their discharge into water containing a wide range of organic ligands. Moreover, the adsorption of these ligands can stabilize the CuO NPs in drinking water treatment plants. Thus, their removal from potable water is important to mitigate the risk to humans. The present study explored the efficacy of the coagulation–sedimentation (C/S) process for the removal of tannic acid (TA)-stabilized CuO NPs using polyaluminum ferric chloride (PAFC) as a coagulant. Moreover, the influence of process conditions (stirring speed) and water chemistry (i.e., pH and ionic strength (IS)) were also investigated to determine their impact on removal. The results showed that stirring speed in the reaction phase significantly affected the removal due to increased flocculation compared with stirring speed in the mixing phase. In addition, pH and IS affect the colloidal stability and removal efficiency of CuO NPs. A relatively better removal performance (<99%) of CuO NPs was found at lower coagulant dosage in the pH range 6–8. The addition of organic ligands reversed the surface charge potential and enhanced the colloidal stability of CuO NPs, resulting in the destabilization of TA-CuO NPs, thereby reducing the optimum PAFC dosage for removal. By contrast, the IS above the critical coagulation concentration decreased the removal efficiency due to inhibition of the ionic activity of PAFC hydrolysate in the aqueous environment. Fourier transform infrared findings of TA-CuO NPs composite flocs suggest that the primary removal mechanism might be mediated via the combined effect of neutralization, complexation as well as adsorption.

## 1. Introduction

Copper oxide nanoparticles (CuO NPs) are detected in aqueous environments due to their enhanced production and usage [1,2,3]. The organic ligands adsorb onto the surface of CuO NPs, thereby increasing their fate and transportation in water [4,5]. Moreover, the ligand-stabilized NPs may find their way into the human body via drinking water treatment plants. Many previous studies reported the hazardous effects of CuO NPs on aquatic species and humans [2,6]. Therefore, it is essential to effectively remove the organic ligand-stabilized CuO NPs from the potable water treatment plants. 

The transportation of NPs in the water can be limited by many processes, i.e., bioremediation, ion exchange, membrane separation, adsorption, coagulation and sedimentation. A few studies reported that metal-based NPs can be controlled via biosorption-activated sludge techniques; however, the removal by bacterial film is significantly affected by the toxicity of nanoparticles [7,8]. The removal of NPs can be achieved via filtration; however, blockage of membrane pores due to NPs increases the treatment cost [9]. Earlier studies confirmed the removal of different metal oxides, including titanium dioxide (TiO_2_), zinc oxide (ZnO), multiwall carbon nanotubes (MWCNT), and CuO NPs from aqueous environment [10]. Coagulation using commercially available polyaluminum chloride (PACl) was not effective in eliminating the NPs from the wastewater of industrial complex [11]. Moreover, the efficient removal of cadmium telluride (CdTe) quantum dots and many commercial NPs was achieved using a combination of alum coagulation as well as membrane filtration [12]. The particle size, surface properties, concentration of NPs, and organic ligands in an aqueous environment also affect the coagulation performance.

Studies reported that the coagulant type and concentration are the primary parameters controlling the coagulation–sedimentation (C/S) performance of metal oxide NPs, i.e., C_60_ and CNTs [11,13]. Iron-based coagulants such as ferric chloride (FC) in comparatively large amounts can remove the Aldrich humic acid (AHA)-coated CNTs [14]. A recent study investigated the removal of HA and surfactant-stabilized CNTs via the combination of alum and PACl coagulant. PACl showed better removal efficiency of CNTs than alum during the C/S process [15]. Factors such as solution pH and ionic strength (IS) affect the colloidal behavior of NPs, as well as the activity of coagulants. IS neutralizes the negative surface potential and efficiently compresses the electrical double layer (EDL) around the particle and thus increases the aggregation of NPs in solution. Bian et al. reported that polydentate organic ligands such as AHA may enhance the dissolution of ZnO NPs at an alkaline (9–11) pH [16]. Moreover, process parameters such as stirring speed in the mixing and reacting phases may also influence the coagulation performance. However, studies exploring the coagulation behavior of organic ligand-stabilized CuO NPs under different hydraulic conditions and water matrices were rarely investigated by environmental scientists. Thus, it is necessary to comprehensively understand the C/S behavior of organic ligand-stabilized CuO NPs in different hydraulic conditions and heterogenous water environments.

Therefore, the purpose of this study was to investigate the influence of process parameters and water chemistry on the elimination of organic ligand-stabilized CuO by PAFC. Tannic acid was used as a model hydrophilic organic ligand. Moreover, various parameters were altered to explore the effect of mixing speeds, pH, and IS during the C/S process.

## 2. Results and Discussion

### 2.1. Characteristics of the Stabilized TA-CuO NP Suspension

**Figure 1:**
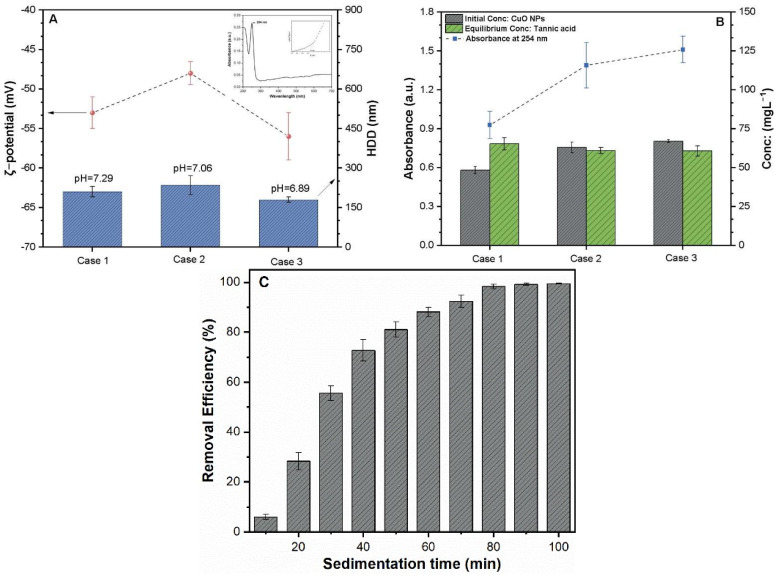
Characteristics of the TA-stabilized CuO NPs suspension. (**A**) ζ−potential and HDD; (**B**) initial and equilibrium concentration of CuO and TA in different groups; (**C**) influence of sedimentation time on the removal efficiency of TA-CuO NP suspensions.

Figure 1A,B illustrates the different characteristics of prepared stock TA-CuO NP suspension in the three experimental groups. The addition of TA substantially decreased the surface potential of CuO NPs, thereby resulting in the formation of a TA-stabilized CuO NP suspension. The slight deviation can be observed in the characteristics of three stock TA-CuO NP suspensions, which might be related to the instability of probe sonication (Figure 1A,B). A significant difference in the initial concentration of CuO NPs (47.8 to 65.10 mg·L^−1^) was noted. In contrast, the equilibrium concentration of TA and pH values remained stable in all three TA-CuO NP (cases 1, 2, and 3) suspensions. The ζ-potential and HDD values of the three suspensions were 53.5 ± 5.5 mg·L^−1^, −48.8 ± 3.7 mV, 57.6 ± 6.3, and 210 ± 5, 235 ± 8, 180 ± 1.5 nm respectively. Moreover, the result of initial experiments revealed that 80 min of sedimentation time was sufficient for the removal of destabilized CuO NPs to attain an equilibrium (Figure 1C). All three stock TA-CuO NPs solutions were semi-diluted with pH adjusted to 7.0 prior to use in different sets of experiments.

### 2.2. Effect of Hydraulic Settings on the Removal Efficiency of TA-CuO NPs

**Figure 2:**
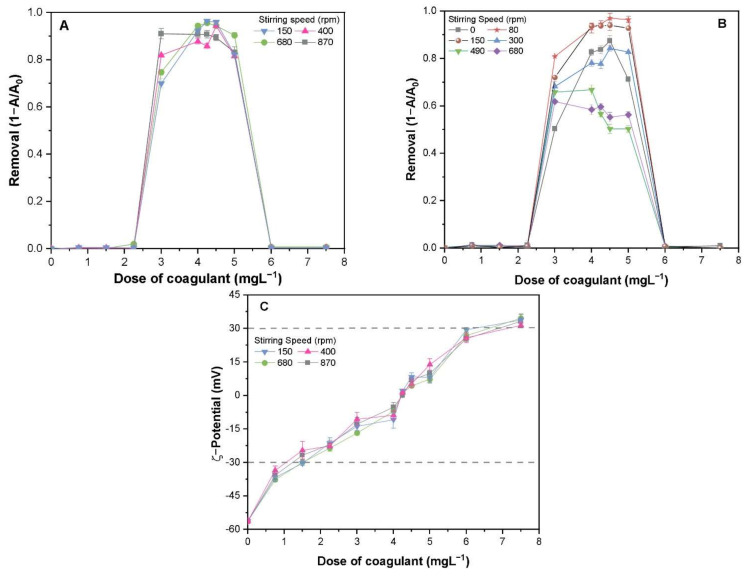
Removal efficiencies of TA-CuO NPs under various stirring speeds and PAFC dosages: (**A**) mixing phase, (**B**) reacting phase, and (**C**) ζ−potential of TA-CuO NPs.

Figure 2A illustrates the influence of stirring speed in the mixing phase on the removal of TA-CuO NP suspension. It was found that altering the stirring speed at the mixing phase had a marginal impact on the removal efficiency, and the removal curves mostly intersected at different points. The range of efficient coagulant concentrations (ECC) was 2.25–6.01 mg·L^−1^, with the highest removal efficiency varying from 90–98%. Furthermore, a slightly enhanced removal was observed (>98%) at a mixing speed of 680 rpm, which was set for the reacting phase experiments. Figure 2B shows that the overall removal efficiency of TA-CuO NPs varied significantly with the change in the stirring speed; however, the ECC range remains unchanged (2.25–6.01 mg·L^−1^). The enhanced removal of around 98% was achieved at the stirring speeds of 80 and 150 rpm, and then investigated at other stirring speeds (300, 490, and 680 rpm). Moreover, it is noteworthy that rapid mixing was not appropriate at 80 rpm. Thus, 150 rpm was selected as the optimal stirring speed in the reacting phase for other sets of experiments. 

In general, the effect of stirring speed in the reacting phase on the removal of TA-CuO NPs was found to be significant when compared with the mixing phase. The stirring speed of the mixing phase primarily affects the mixing intensity of the coagulant, thereby leading to the formation of CuO NP colloids even at a low stirring speed. Altering the stirring speed during the mixing phase resulted in significant variation in the removal efficiencies, but the same speed in the reacting phase might affect the flocculation and the overall removal performance. At a low stirring speed, tiny CuO NP aggregates formed during the mixing phase were unable to collide with other NPs to produce large and dense flocs, while high stirring speeds generated high-strength shearing forces, which destroyed the flocs formed. The removal rate in both conditions was less than moderate. Our results are consistent with a previous study, which reported the increased removal of red dye at moderate stirring speed using iron salts and alum sulfate [17].

To better understand the removal phenomenon, the ζ-potential of TA-CuO NPs was determined under both process conditions. It was observed that the surface potential of TA-stabilized CuO NP suspension was steadily enhanced (−56.05 to 38.7 mV) by raising PAFC concentration from (0 to 8 mg·L^−1^) as shown in Figure 2C. The effect of change in the stirring speed in the mixing phase had insignificant influence on the surface charge of TA-CuO NPs, implying negligible interaction among the NPs and PAFC (Figure 2A,C). The ζ-potential of TA-CuO NPs in the ECC range (ca. 2.25–6.01 mg·L^−1^) was between −30 and 30 mV. In this range, the maximum removal of TA-CuO NPs was achieved due to weak electrostatic repulsion forces among the collides, which was consistent with another study [16]. After optimal dosage, the higher PAFC concentration resulted in enhanced ζ-potential above 30 mV, thereby decreasing the removal of TA-CuO NPs due to the strong electrostatic hindrance [18]. Consequently, the observed removal curves (Figure 2A,B) showed a distinct inverted “U” shape. In general, higher and lower dosages of PAFC resulted in lower removal, suggesting the need for an optimum coagulant dose to ensure the effective coagulation of TA-stabilized NPs suspension.

### 2.3. Influence of Solution pH on the Stability of TA-CuO NPs

**Figure 3:**
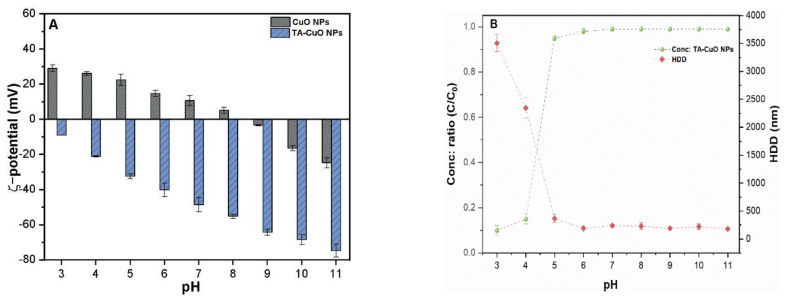
Under different solution pH levels: (**A**) ζ-potential of CuO NPs with and without TA; (**B**) colloidal stability and HDD of the TA-CuO NPs. The concentration ratio C/C_0_ indicates the colloidal stability of TA-CuO NP suspension.

The colloidal stability of metal-based NPs in an aquatic environment largely depends upon the pH of the receiving body. As shown in Figure 3A, the addition of TA to CuO NPs reverses the ζ-potential towards the negative trajectory. The TA-CuO NP suspension remained dispersed and stable at pH values of 5–11, with an approximate HDD value of 280 nm. Figure 3B shows that TA-CuO NPs rapidly sedimented between pH 3 and 4, with enhanced HDD. The increased rate of precipitation of the TA-CuO NPs under acidic pH conditions might be related to enhanced settling of colloids due to H-bonding and/or polar interaction among the CuO-adsorbed organic ligands without effective electrostatic repulsion. A recent study reported a similar pH-dependent precipitation of multiwall carbon nanotubes in the presence of tannic acid [19]. Therefore, to minimize this effect in the experiment, the pH of the TA-CuO NP suspension was adjusted to between 5 and 10.

### 2.4. Influence of Solution pH on the Removal of TA-CuO NP Suspension

**Figure 4:**
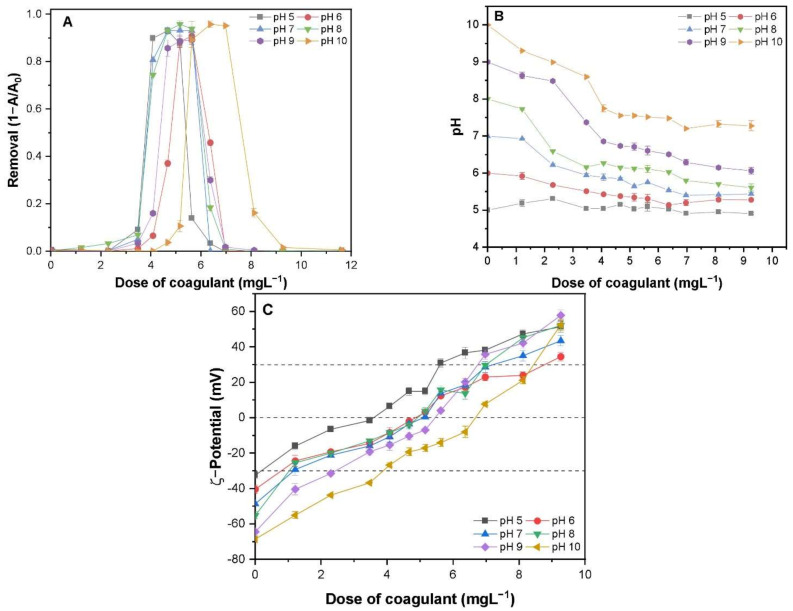
Effect of various PAFC dosages and solution pH on (**A**) removal efficiency; (**B**) final pH of suspension, and (**C**) ζ-potential of TA-CuO NPs.

The removal rate of TA-CuO NPs under different levels of solution pH is presented in Figure 4A. The coagulation curve continued to remain in an almost reverse “U” pattern at various pH conditions. However, a slight forward shift in the curve was observed with increasing suspension pH (Figure 4A). The removal efficiencies at various pH conditions remained the same; however, the effective PAFC dosage gradually increased from 2.25 to 5.75 mg·L^−1^ at pH 5 to 6.05–10.95 mg·L^−1^ at initial pH 10 (Figure 4A). These results suggest that the coagulation of TA-CuO NPs was feasible within the broad pH range and enhanced the removal performance with a lower coagulant dose at low pH values. The addition of PAFC coagulant lowered the solution pH, especially at high initial pH conditions, due to the buffer effect of Al^3+^ and Fe^3+^ ions (Figure 4B). The decrease in pH value might lower the negative surface potential of TA-CuO NPs; however, it does not reverse the surface charge from negative to positive (Figure 3A). The high dosage of PAFC can oversaturate the negative TA-CuO NPs and positively charged colloids [20]. The ζ-potential was increased with increasing PAFC concentration in the solution (Figure 4C). Moreover, the range of weak electrostatic repulsion (±30 mV) was enhanced with increasing suspension pH. These results suggest the identical behavior of repulsive forces with the ECC range of individual suspension pH (Figure 4A).

In addition, the suspension pH may control the coagulation behavior by altering the surface potential of NPs, product species of PAFC and their equilibrium hydrolysis [21]. The primary species of Al and Fe in PAFC solution include the monomer (Al_a_), polymer (Al_b_ or Al_13_), colloidal form (Al_c_), and Fe^3+^, respectively [21]. The concentration of polymer (Al_b_ or Al_13_) species was enhanced under the acidic/neutral environment and significantly decreased with increasing solution pH towards the alkaline zone. In contrast, the behavior of Al_a_ species with suspension pH was opposite to that of polymer and colloidal form [22]. Moreover, Fe(III) at neutral pH facilitated the C/S process as a substantial amount of FeOOH was produced. The positive charges of Al_13_ and Fe(III) species exhibit strong neutralizing as well as adsorbing capability, thereby removing the TA-CuO via coagulation [21]. The studied TA-CuO NPs suspension pH values range from 5.0 to 10. However, an increase in the dosage of PAFC resulted in a decrease of final pH towards the acidic range (5.03–8.70) Figure 4B. The hydrolysis of PAFC may have influenced the coagulation behavior during the removal of organic ligand-stabilized CuO NPs. Moreover, the removal efficiency was similar under different pH values used.

### 2.5. Influence of Electrolyte Concentration on the Stability and Removal of TA-CuO NPs

**Figure 5:**
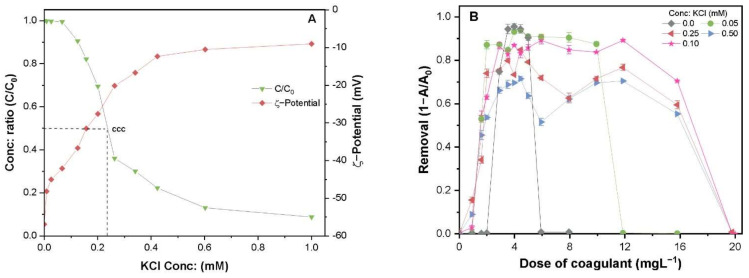

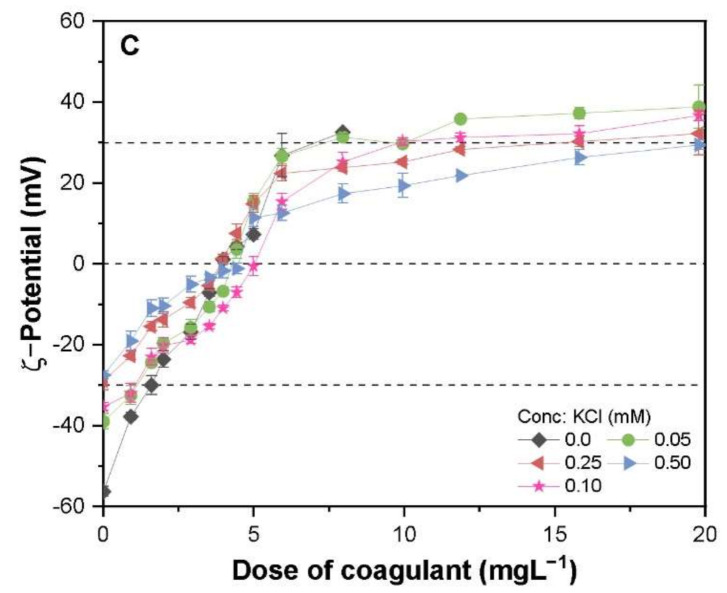
Effect of various KCl concentrations on TA-CuO NPs (**A**) stability (C/C_0_); (**B**) removal efficiency by PAFC, and (**C**) corresponding ζ-potential at various dosages of PAFC.

The addition of monovalent electrolyte KCl greatly decreased the negative charge potential of TA-CuO NPs, thereby destabilizing colloidal suspension (Figure 5A). Destabilized TA-CuO NPs exhibited a standard precipitation curve against the IS of KCl. The critical coagulation concentration (CCC) of KCl for TA-CuO NPs was calculated using Derjaguin–Landau–Verwey–Overbeek (DLVO) theory [23]. The CCC can be defined as the minimum amount of an electrolyte required to destabilize NP suspension completely. The value of CCC provides essential information about NPs stability and can thus be used to predict the fate and transport of NPs in natural waters. The CCC of KCl was found to be 0.225 mM, where more than 50% of the suspended CuO NPs destabilize. Figure 5B illustrates the removal curves of TA-CuO NP suspension under different IS. It can be observed that the incorporation of KCl changes the maximum removal efficiency and ECC range of PAFC. The removal efficiency remained constant at concentrations below the CCC of KCl; however, it was substantially decreased when the concentration was above CCC. The enhanced IS significantly increased the effective PAFC concentration range and lowered the required PAFC dosage to achieve the highest removal efficiency (Figure 5B). Thus, a lower concentration of monovalent salt ions improved the coagulation performance. In contrast, the high IS may hinder the ionic activity of the charged species of Al/Fe (AlO_4_Al_12_(OH)_24_(H_2_O)_12_)^7+^, Fe(OH)_2_), thus decreasing the removal efficiency. A lower removal rate of hydrophobic organic ligand at high IS was reported in a previous study [22]. At optimum concentration, the electrolyte efficiently compresses the EDL around the TA-CuO NPs and reduces the absolute surface charge (−30 mV to +30 mV) of TA-CuO NP suspension under different concentrations of PAFC (Figure 5). Moreover, the range of coagulant concentrations was enhanced with increased IS of solution and showed a similar ECC range of individual IS (Figure 5B,C). The increased removal in these conditions might be attributed to the weak electrostatic hindrance effect, enhancing the forces of attraction among NPs.

### 2.6. Effect of TA-Stabilized CuO NP Concentration on the Removal Process

**Figure 6:**
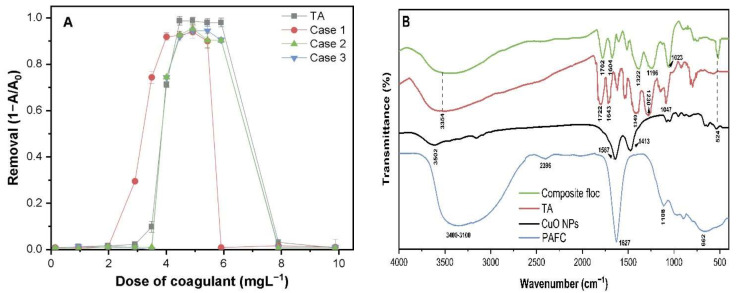
(**A**) Removal efficiencies of 3 sets of TA-CuO NP suspensions and the dissolved TA by PAFC; (**B**) FTIR spectra of pristine chemicals, i.e., CuO-NPs, PAFC, TA and composite floc obtained at optimum dose.

Figure 6A illustrates the removal patterns of the three sets of diluted stock suspension of TA-CuO NPs obtained in previous experiments. It is noteworthy that the pattern of curves and removal efficiency remains constant, whereas the ECC range shifted forward with an enhanced concentration of TA-stabilized CuO NPs suspension. A higher dose of coagulant was required to achieve a similar removal efficiency for the TA-CuO NP suspension containing a higher concentration of CuO NPs. For instance, in case 1, the required PAFC dosage was about 3.12 mg·L^−1^ to achieve 78% removal of TA-stabilized CuO NPs containing 23.9 mg CuO NPs. However, the added concentration of PAFC was 4.07 mg·L^−1^ to achieve a similar removal efficiency of ligand-stabilized CuO NPs at 30.1–31.6 mg CuO NPs/L. The coagulation curves of TA suspension (40 mg·L^−1^) at neutral pH also showed the same inverted “U” pattern (Figure 6A). The removal efficiency and ECC range of TA suspension were higher compared with TA-CuO NP solutions. These findings strongly suggest the interaction between PAFC and ligand molecules via H-bonding and other associated groups present in TA. 

The FTIR spectra of pristine chemicals including CuO, TA, PAFC, and composite flocs obtained after the C/S process were analyzed to elucidate the probable removal mechanism, as illustrated in Figure 6B. The broad spectrum peaks around 3500–3100 cm^−1^ were attributed to OH stretching and (Al/Fe–OH) vibration frequency [23]. The peaks observed around 1722, 1643, 1230, and 1047 cm^−1^ were associated with stretching vibrations of C=O, aromatic C=C, phenolic C–O, and C–O(H), respectively. In contrast, the peaks at 662 and 524 cm^−1^ were ascribed to the bending vibration of Fe–OH–Fe and Cu-O vibration frequency, respectively. Organic ligands such as TA contain many OH functional groups in their steric structure, which prevent the interaction between moieties with the surface of CuO NPs and others act as functional groups.

The IR spectra of composite floc (TA-CuO + PAFC) obtained after coagulation show a significant shift in major groups such as C=O, C–O and C=C along with minor variations in the associated groups (Figure 6B). For example, the C=O stretching band around 1722 cm^−1^ was decreased and shifted to lower wavenumbers at around 1702 cm^−1^, thereby confirming the strong inner-sphere complexation with free metal ions. Furthermore, a significant shift occurred in the C-O (1349 cm^−1^ to 1322 cm^−1^) bond stretching vibration with decreased intensity, suggesting the interaction between Fe^3+^ and the phenolic hydroxyl group of TA during coagulation [24]. Few spectral peaks in composite floc around 1600 to 1000 cm^−1^ were attributed to the complexation of ligand molecules and Al/Fe ions. These findings are consistent with the study of Zhao and Liu, which reported that OH groups in TA facilitate the formation of metal complexes [25]. Moreover, the Fe ions may form complexes with (−C–O) via two adjacent hydroxyls (catechols), while the co-existence of a third adjacent hydroxyl (pyrogallols) enhanced the stability of the formed complex [26]. The major changes and increased strength of certain bands in composite flocs facilitated the complex reaction between metal ions and PAFC in ligand-containing waters. Thus, based on the FT-IR spectrum, the potential removal mechanism of TA-CuO NPs might involve a combination of charge neutralization, inner-sphere complexation, and adsorption. 

## 3. Materials and Methods

### 3.1. Materials

The selected CuO NPs (Sigma Aldrich, St. Louis, MO, USA) with supplier-reported characteristics (purity > 99.0%, mean outer diameter < 50 ± 6 nm) were used without further purification. The coagulant polyaluminum ferric chloride (PAFC) containing 26% of Al/Fe was purchased from water treatment material company Gongyi Tenglong Co., Ltd., Qingdao, China. Tannic acid (Sigma Aldrich, USA) was preferred to reflect the relatively hydrophilic organic ligands with a molecular weight of about 1700 Da (4).

### 3.2. Stock Solution

TA stock suspension was prepared by weighing 100 mg of TA powder and dissolved in 100 mL of ultrapure Milli-Q water. The suspension pH was adjusted to neutral with 100 mM HCl to completely dissolve the organic ligand. The CuO NPs (200 mg·L^−1^) were probe-sonicated (40 kHz, 400 W, 30 min) in TA (50 mg·L^−1^) suspension. Subsequently, the obtained ligand-stabilized CuO NP suspension was further centrifuged at 3200 rpm for 40 min and then stored in the dark. Prior to the experiment, the concentration of dissolved TA in stabilized CuO NPs suspension was analyzed with total organic carbon (TOC) analyzer (TOC-VCPH, Shimadzu, Japan). Moreover, the concentration of CuO NPs was also calibrated by measuring the absorbance at 254 nm. The stock solution 50 mg·L^−1^ of commercially available PAFC coagulant (26% Al content) was prepared by directly dissolving into the ultrapure Milli-Q water.

### 3.3. Coagulation Experiments

The coagulation–sedimentation-flocculation experiments were performed in a jar test apparatus with six automatic lifting mixers (Model: SJ-10, Young Hana Tech Co., Ltd., Gyeongsangbuk-Do, Korea). The ligand-stabilized CuO NP suspension was diluted to the desired concentration prior to the jar test experiments. In addition, pH was adjusted to pre-determined level before coagulation experiments. In short, at the beginning of rapid mixing (mixing phase), 5 mL of PAFC and 45 mL of TA-stabilized CuO NP suspension were transferred to 100 mL glass beakers. The rapid mixing was sustained for 1 min and the suspension was then slowly mixed (reacting stage) for 20 min. The mixture was left to sediment for 100 min, followed by the analysis of the collected supernatants to determine the final solution pH and turbidity at 254 nm (UV-vis absorbance).

### 3.4. Experimental Matrices

Following the above procedure, three sets of experiments were designed to investigate the influence of various parameters, i.e., hydraulic condition, solution pH, and IS. In the first set of trials, the effects of hydraulic setting on the removal efficiency were analyzed at different stirring speeds in the mixing and reacting phases. The stirring speed in the reacting phase was set at 150 rpm to investigate the effect of the stirring speed during the mixing phase. Next, the effect of the stirring speed on the reacting phase was determined and the stirring speed in the mixing phase was set at 680 rpm. Subsequently, the mixing phase, the surface charge and the hydrodynamic diameter (HDD) of the NPs were examined. In the remaining two sets of C/S experiments, the influence of different solution pH (5–11) and IS values (0–1.0 mM) of TA-CuO suspension were investigated. The pH and IS of the solution were adjusted using NaOH, HCl, and KCl, respectively. In both sets of trials, the stirring speeds in the rapid mixing and reacting phases were set up at 680 and 150 rpm, respectively. Moreover, under similar experimental conditions in the absence of CuO NPs, the removal efficiency of TA solution (40 mg·L^−1^) by PAFC was determined. The removal efficiency of CuO NPs was calculated by measuring the absorbance at 254 nm before and after the coagulation experiment. 

### 3.5. Analytical Procedure

The residual concentration of CuO NPs was determined by measuring the absorbance at 254 nm with a UV-Vis spectrophotometer (Optizen, 2120 UV-Vis, Mecasys, Korea). A zetasizer (Nano-ZS90, Malvern, UK) was used to measure the Zeta potential and the HDD of NPs. In addition, to elucidate potential removal mechanisms, Fourier transform infrared analysis (FT/IR-4700, JASCO Analytical Instruments, Easton, PA, USA) in the range of 400 to 4000 cm^−1^ was conducted before and after the C/S experiments. Each experiment was repeated three times and the relative standard deviations (STD) were recorded as <5%.

## 4. Conclusions

In the present work, we explored the effect of hydraulic settings and water chemistry on the removal of TA-stabilized CuO NPs via PAFC coagulation. The stirring speed in the reacting phase affects the development and shape of precipitated floc by altering the shear forces, thus affecting the overall efficiency. Lower pH and high IS conditions may facilitate the destabilization and decrease of TA-CuO NPs and thereby improve the removal efficiency. In contrast, high IS (above CCC) minimizes the removal efficiency due to inhibition of the activity of PAFC hydrolysate. The discrepancy in the initial mass of TA-CuO NPs observed affects the required coagulant dose and removal rate of NPs. 

The FT-IR study of composite flocs indicated the role of primary mechanisms, i.e., charge neutralization, complexation, and adsorption, in the removal of TA-CuO NPs via coagulation. Overall, the results provide insight into the removal behavior of ligand-stabilized CuO NPs from heterogeneous water environments via the coagulation process.

## Data Availability

The data presented in this study are available on request from the corresponding author.

## Sample Availability

Not available.
